# Supplementary material for: Tumour heterogeneity and intercellular networks of nasopharyngeal carcinoma at single cell resolution
Source: Nat Commun. 2021 Feb 2;12:741. doi: 10.1038/s41467-021-21043-4 (PMC7854640; doi:10.1038/s41467-021-21043-4)
Supplement: Supplementary file 1 — Supplementary Information [file 41467_2021_21043_MOESM1_ESM.pdf]

Supplementary Figure 1

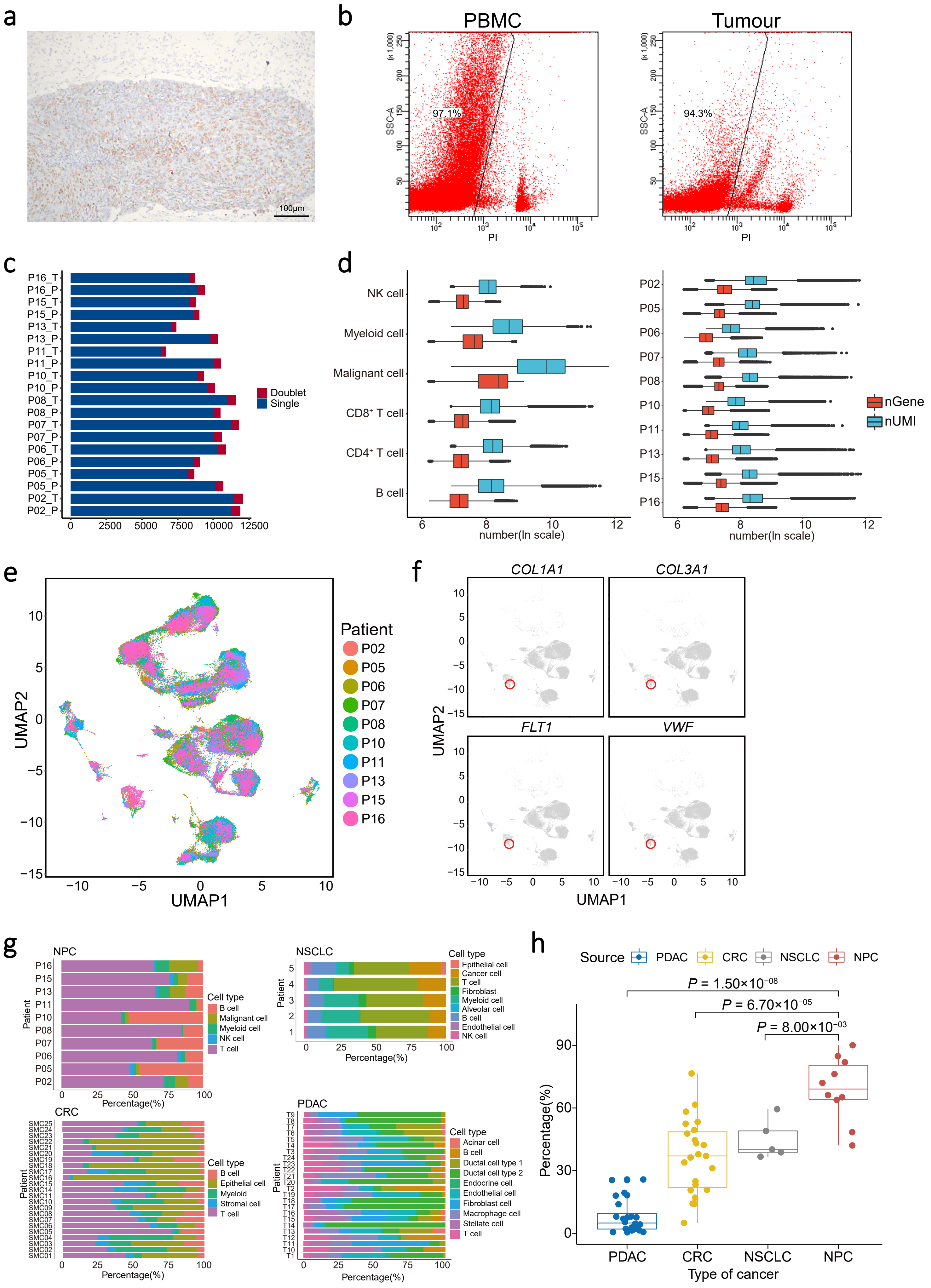

Supplementary Fig. 1 Basic information of the single cell RNA sequencing and TCR sequencing data.

a Representative image of the *in-situ* hybridization of EBERs in NPC biopsy section. Brown staining indicates scattered EBER-positive malignant cells in NPC tissue. Images are representative of ten patients. Scale bars, 100 μm.

b Representative images of flow cytometry sorting for viable cells from PBMC (left panel) and tumour tissues (right panel) of ten patients. Cells were stained with PI (indicating apoptotic cells or fractions). All samples were gated on singlets and PI.

c Bar plot showed the number of single (dark blue) and doublet (dark red) cells in NPC as evaluated using by R packages "DoubletFinder". The naming system was as follow, taking an example of "P02_P" and "P02_T" representing PBMC and tumour tissues of the patient P02, respectively.

d Box plots showed the number of UMIs and genes for cell types (left panel) and patients (right panel; ln scale as defined in the inset; n = 176,447). Endpoints depict minimum and maximum values; centre lines denote median values; whiskers denote 1.5 × the interquartile range; black dots denote each patient. Cell clusters (left panel) and patients (right panel) are indicated at the y-axis, and the numbers of interactions are indicated at the x-axis.

e UMAP plot of the overall 176,447 cells, with each cell colour coded for the patient of origin.

f UMAP plots showed the normalized expression of markers for fibroblasts (*COL1A1* and *COL3A1*) and endothelial cells (*FLT1* and *VWF*). Each dot represents a single cell, and the depth of colour from grey to blue represents low to high expression.

g Bar plots showed the proportion of each cell type in NPC, NSCLC (non-small-cell lung cancer), CRC (colorectal cancer), and PDAC (pancreatic ductal adenocarcinoma). Each bar represents an individual patient, with cell proportions coded as different colours for cell types on the right.

h Box plots showed the proportion of T cell in the TME of NPC (n = 10), NSCLC (n = 5), CRC (n = 23), and PDAC (n = 24). Comparison was made by using two-sided Wilcoxon test. The types of cancer and the proportions of T cells are indicated at the x- and y-axis, respectively. Endpoints depict minimum and maximum values; centre lines denote median values; whiskers denote 1.5 × the interquartile range; coloured dots denote each patient.

Supplementary Figure 2

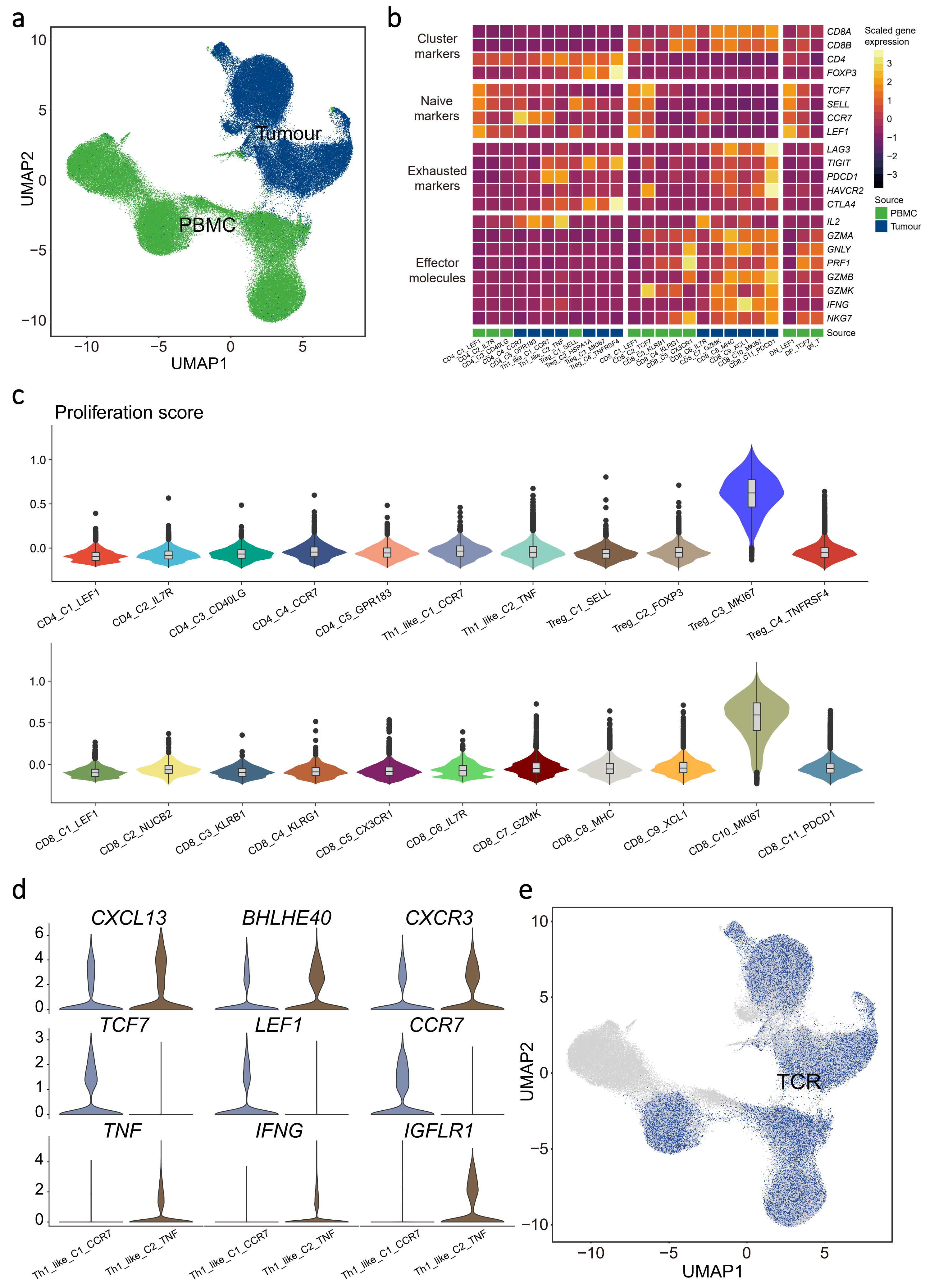

Supplementary Fig. 2 Expression profile of T cells.

a UMAP plot showed 141,875 T and NK cells in NPC. Each dot represents a cell, coloured according to their origins from tumour or PBMC.

b Heatmap showed the normalized mean expression of signature genes (rows) for each T cell cluster (columns). Filled colours from dark blue to yellow represent scaled expression levels from low to high. The origins of T cell clusters from tumour (blue) or PBMC (green) were indicated as rectangles at the bottom.

c Violin plots showed the proliferation scores of each $CD4^+$ (top panel; n = 47,384) and $CD8^+$ (bottom panel; n = 62,244) T cell clusters. Box plots inside the violins indicated the quartiles of corresponding score levels, with horizontal lines representing median values and whiskers extend to the farthest data point within a maximum of 1.5 × the interquartile range. Cell clusters and the proliferation scores are indicated at the x- and y-axis, respectively.

d Violin plots showed the normalized expression of signature genes of $CCR7^+$ and TNF^+ Th1-like cell clusters (n = 5,805). In each plot, cell clusters and the expression level of a gene as the chart tile are indicated at the x- and y-axis, respectively.

e UMAP plot showed the distribution of T cells with detected TCR clonotypes. Each dot represents a single cell, coloured with blue or grey for T cell without detectable TCR or non-T cell, respectively.

Supplementary Figure 3

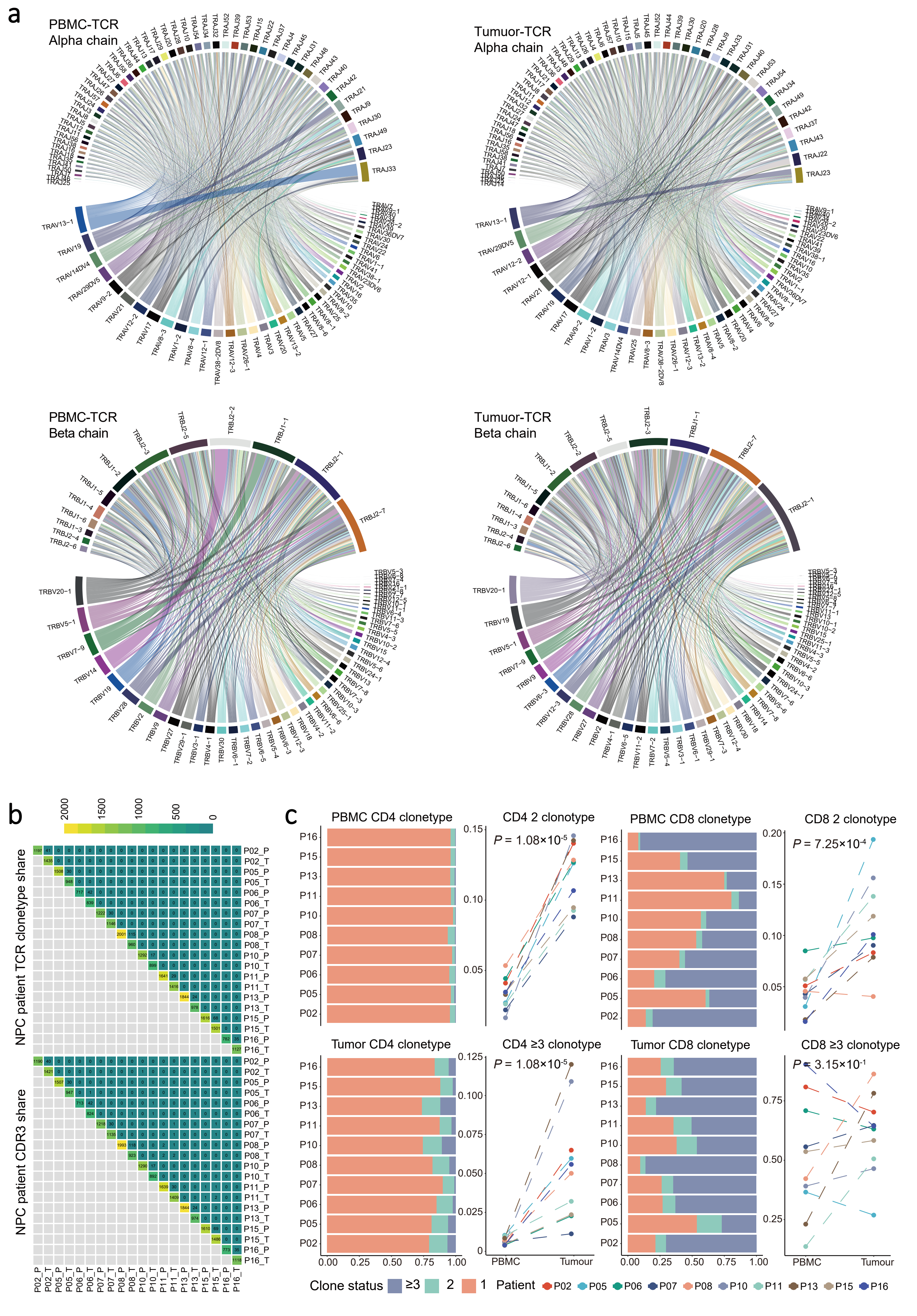

Supplementary Fig. 3 TCR repertoire of T cells between peripheral blood and tumour tissues.

a Chord diagrams showed the pairing of V and J genes in alpha (top panels) and beta (bottom panels) chains of TCR in PBMC (left panels) and tumour (right panels). Ribbons connecting chains indicate the frequency of the pairing, coloured according to genes. V genes on the diagrams were ordered clockwise according to their frequencies from low to high, as were J genes.

b The sharing of TCR clonotypes (top panel) and CDR3s (bottom panel) between peripheral blood and tumour tissues across all 10 patients. Filled colours from green to yellow represent the sharing numbers from low to high. The naming system was as follow, taking an example of "P02_P" and "P02_T" representing PBMC and tumour tissues of the patient P02, respectively.

c Bar plots showed the percentage of single, double and multiple TCR clonotypes across $CD4^+$ T cells (left panel) or $CD8^+$ T cells (right panel) between peripheral blood and tumour, coloured according to clone status. Scatter plots showed the difference in double and multiple TCR clonotypes across $CD4^+$ T cells (left panel) or $CD8^+$ T cells (right panel) between peripheral blood and tumour, coloured according to each patient. *P* value was calculated using two-sided Wilcoxon test.

Supplementary Figure 4

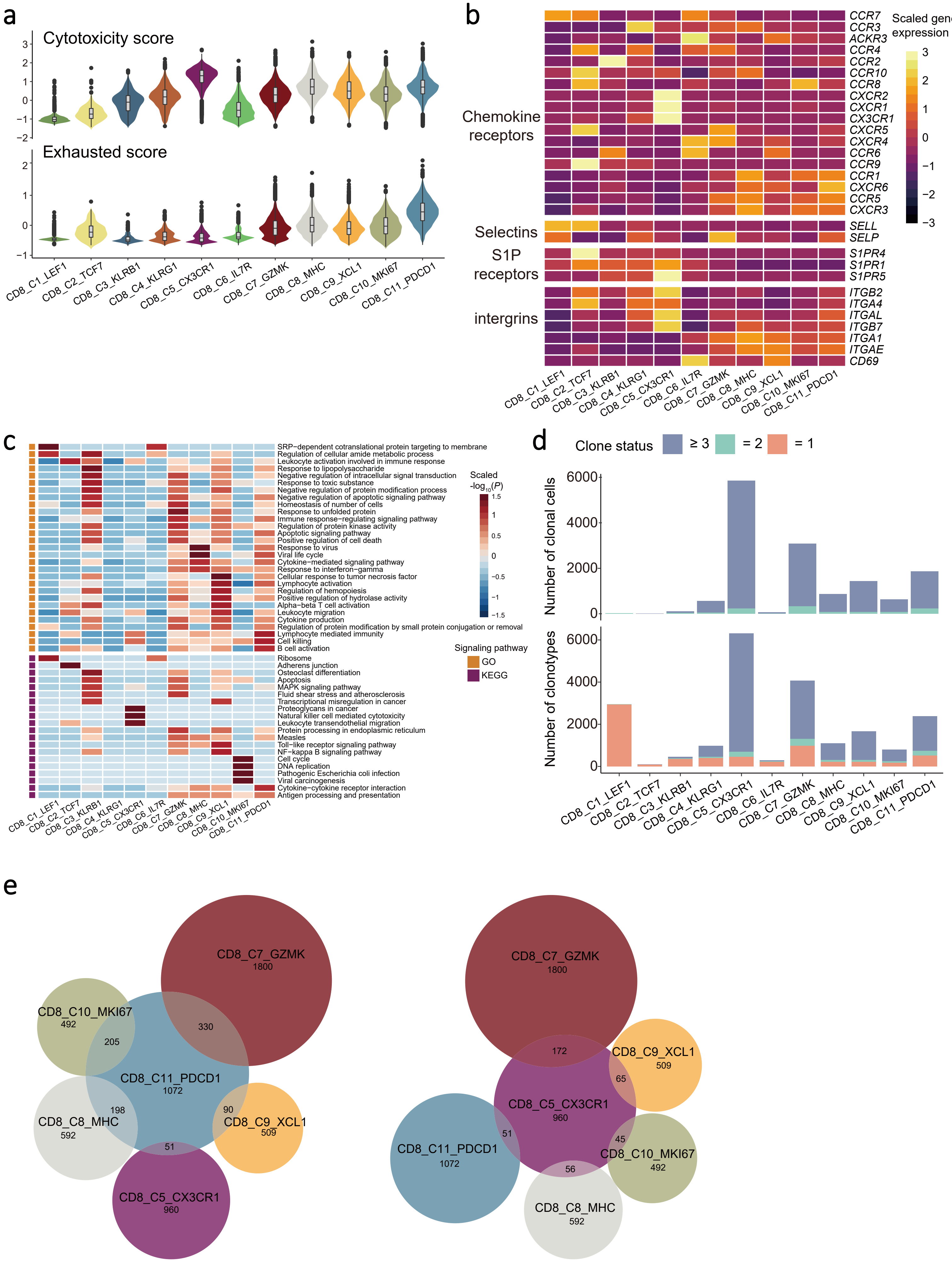

Supplementary Fig. 4 Expression profiles and TCR clonotypes of $CD8^+$ T cells.

a Violin plot showed the cytotoxicity (top panel) and exhausted (bottom panel) scores of each $CD8^+$ T cell cluster (n = 62,244). Box plots inside the violins indicated the quartiles of corresponding score levels. Endpoints depict minimum and maximum values; centre lines denote median values; whiskers denote 1.5 × the interquartile range; black dots denote each cell. Cell clusters and the signature scores are indicated at the x- and y-axis, respectively.

b Heatmap showed the normalized mean expression of migration-related genes (rows) of each $CD8^+$ T cell cluster (columns). Filled colours from dark blue to yellow represent scaled expression levels from low to high. Genes are listed as three different groups as indicated.

c Heatmap showed the selected signalling pathways (rows) with significant enrichment of GO and KEGG terms for each $CD8^+$ T cell cluster (columns). Filled colours from blue to red represent scaled expression levels ($-\log_{10}P$ values) from low to high. *P* values were calculated by one-sided hypergeometric test and adjusted for multiple comparisons. Orange and purple squares on the left represent the signalling pathways derived from GO or KEGG analysis, respectively.

d Bar plots showed the number of clonal cells (top panel) and clonotypes (bottom panel) in $CD8^+$ T cell clusters. Bars were coloured according to the clone status as indicated on top. Cell clusters and the numbers are indicated at the x- and y-axis, respectively.

e Venn diagram showed overlapped TCR clonotypes between exhausted $CD8^+$ T cells (CD8_C11_PDCD1) with other $CD8^+$ T cells (left panel) and between $CX3CR1^+CD8^+$ T cells (CD8_C5_CX3CR1) with other $CD8^+$ T cells (right panel). Numbers in the circles and the sharing areas indicate the total numbers and the overlapped numbers of TCR clonotypes, respectively.

Supplementary Figure 5

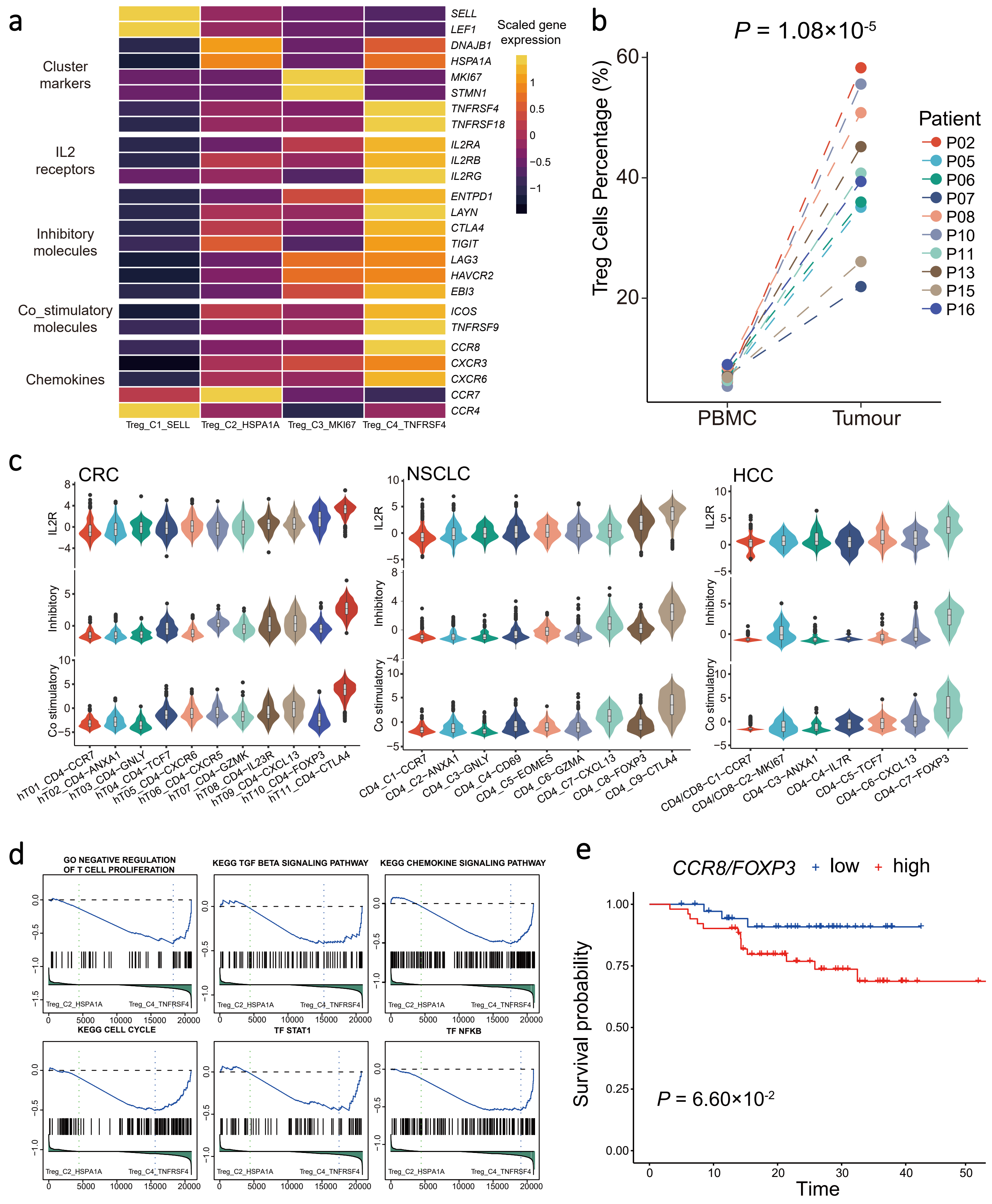

Supplementary Fig. 5 Expression profiles of $CD4^+$ T cells.

a Heatmap showed the normalized mean expression of Treg cell marker and function related genes (rows) for each Treg cell cluster (columns). Filled colours from dark blue to yellow represent scaled expression levels from low to high.

b The percentage of Treg cells in $CD4^+$ T cells from peripheral blood and tumour tissues across 10 NPC patients. Points were coloured according to each patient. *P* value was calculated using two-sided Wilcoxon test.

c Violin plots showed the functional status of $CD4^+$ T cell cluster in CRC (colorectal cancer, n = 3,143; left panel), NSCLC (non-small-cell lung cancer, n = 5,250; middle panel), and HCC (hepatocellular carcinoma, n = 1,076; right panel). Box plots inside the violins indicated the quartiles of corresponding score levels. Endpoints depict minimum and maximum values; centre lines denote median values; whiskers denote 1.5 × the interquartile range; black dots denote each cell. The functional status was determined using the IL2R, inhibitory, and co-stimulatory scores (indicated at the y-axis) of each $CD4^+$ T cell cluster as indicated at the x-axis.

d GSEA analyses revealed the pathway activities (on top of each box) between Treg_C4_TNFRSF4 and Treg_C2_HSPA1A.

e Kaplan-Meier progression-free survival curves of NPC patients. A total of 88 patients with prognostic information were stratified according to the ratio of normalized *CCR8*/*FOXP3* expression. Survival duration and probability were indicated at the x- and y-axis, respectively. *P* value was calculated using two-sided log-rank test.

Supplementary Figure 6

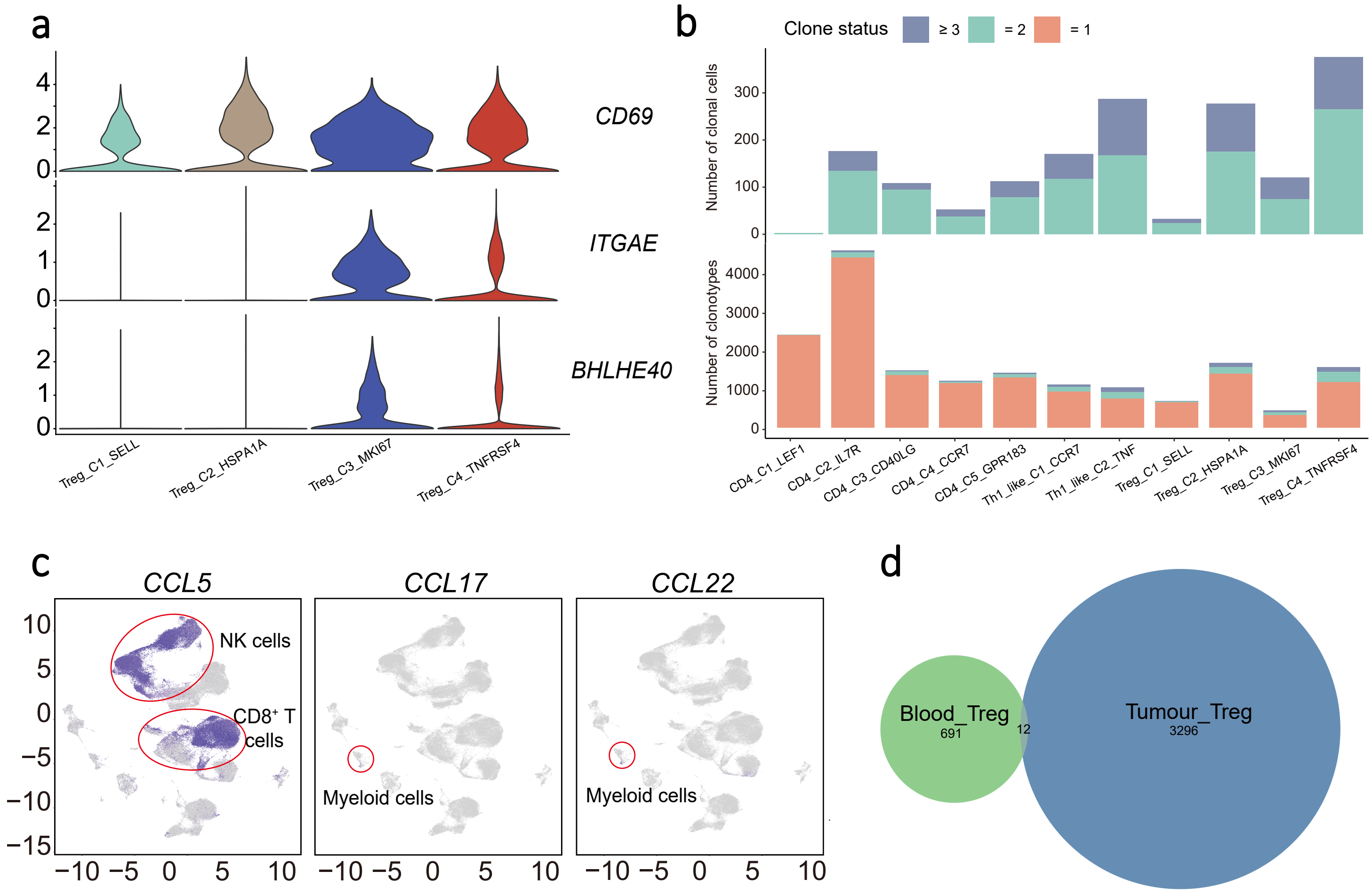

Supplementary Fig. 6 The tissues resident makers and TCR clonotyeps of CD4⁺ T cells.

a. Violin plots showed the normalized expression of tissue-resident markers (*CD69*, *ITGAE*, and *BHLHE40*) of each Treg cell cluster. In each plot, cell clusters and the expression level of a gene as the chart title are indicated at the x- and y-axis, respectively.

b. Bar plots showed the number of clonal cells (top panel) and clonotypes (bottom panel) in CD4⁺ T cell clusters. Bars were coloured according to clone status as indicated on top. Cell clusters and the numbers are indicated at the x- and y-axis, respectively.

c. UMAP plots showed the normalized expression of chemokine ligands (*CCL5*, *CCL17*, and *CCL22*) of 176,447 cells. Each dot represents a single cell and the depth of colour from grey to blue represents low to high expression.

d. Venn diagram showed overlapped TCR clonotypes between peripheral blood and tumour infiltrating Treg cells. Numbers in the circles and the sharing areas indicate the total numbers and the overlapped numbers of TCR clonotypes.

Supplementary Figure 7

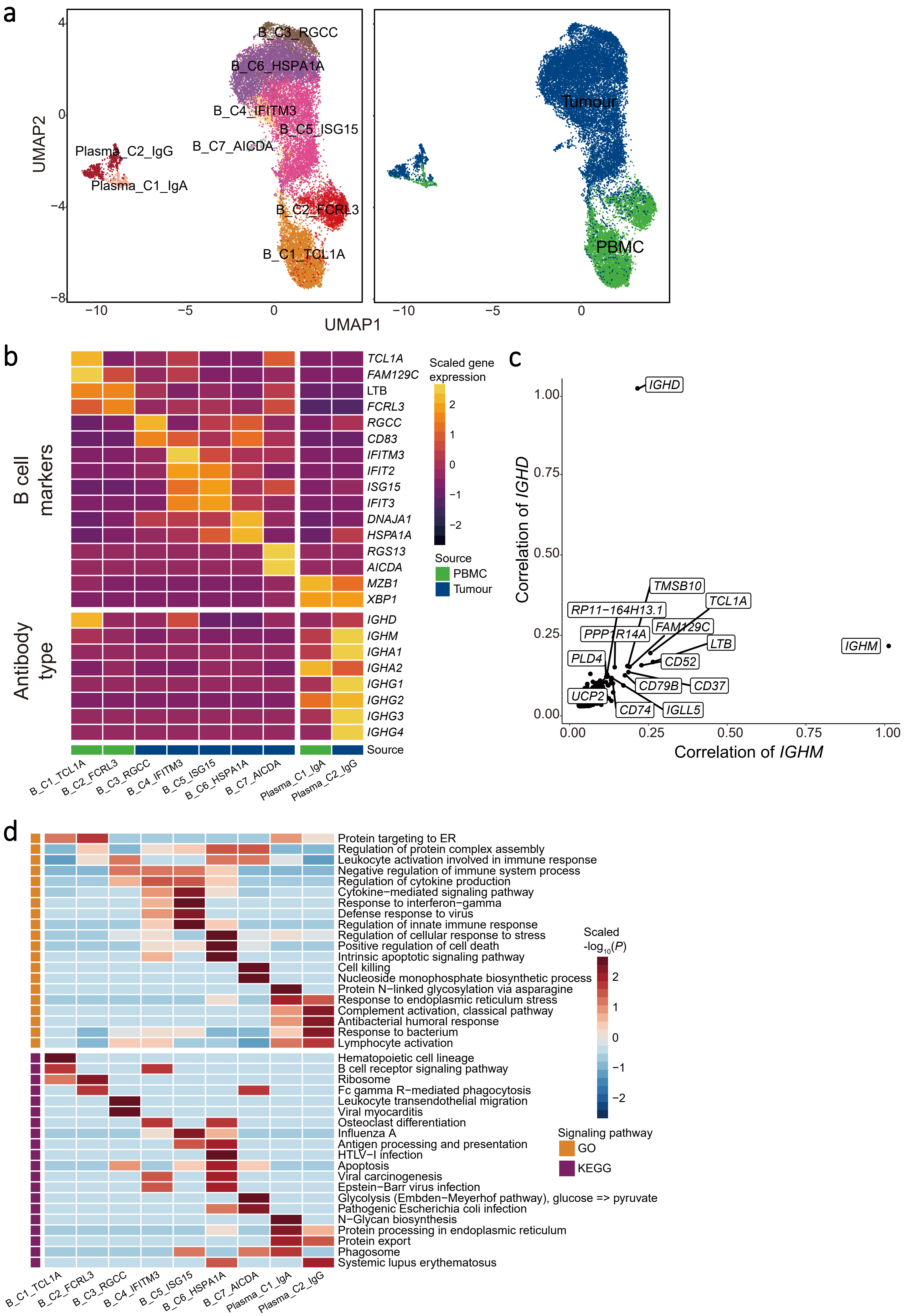

Supplementary Fig. 7 The characteristics of B cell clusters in peripheral blood and tumour tissues.

a UMAP plots of 22,892 B cells clustered into 10 cell types (left panel) from tumour or PBMC (right panel). Each dot represents a cell, coloured according to cell type (left panel) or origin (right panel).

b Heatmap showed the normalized mean expression of B cell marker and antibody type genes (rows) for each B cell cluster (columns). Filled colours from dark blue to yellow represent scaled expression levels from low to high. Cell clusters were indicated at the bottom, with their origins from PBMC or tumour indicated above in green or blue, respectively.

c Scatter plot showed the genes with significant correlation with *IGHM* and *IGHD* in naive B cells (B_C1 and B_C4). Selected signature genes were indicated in black boxes. The Pearson correlation coefficients with *IGHM* and *IGHD* are indicated at the x- and y-axis, respectively.

d Heatmap showed the selected signalling pathways (rows) with significant enrichment of GO and KEGG terms for each B cell cluster (columns). Filled colours from blue to red represent scaled expression levels ($-\log_{10}P$ values) from low to high. P values were calculated by one-sided hypergeometric test and adjusted for multiple comparisons. Orange or purple squares on the left represent the signalling pathways derived from GO or KEGG analysis, respectively.

Supplementary Figure 8

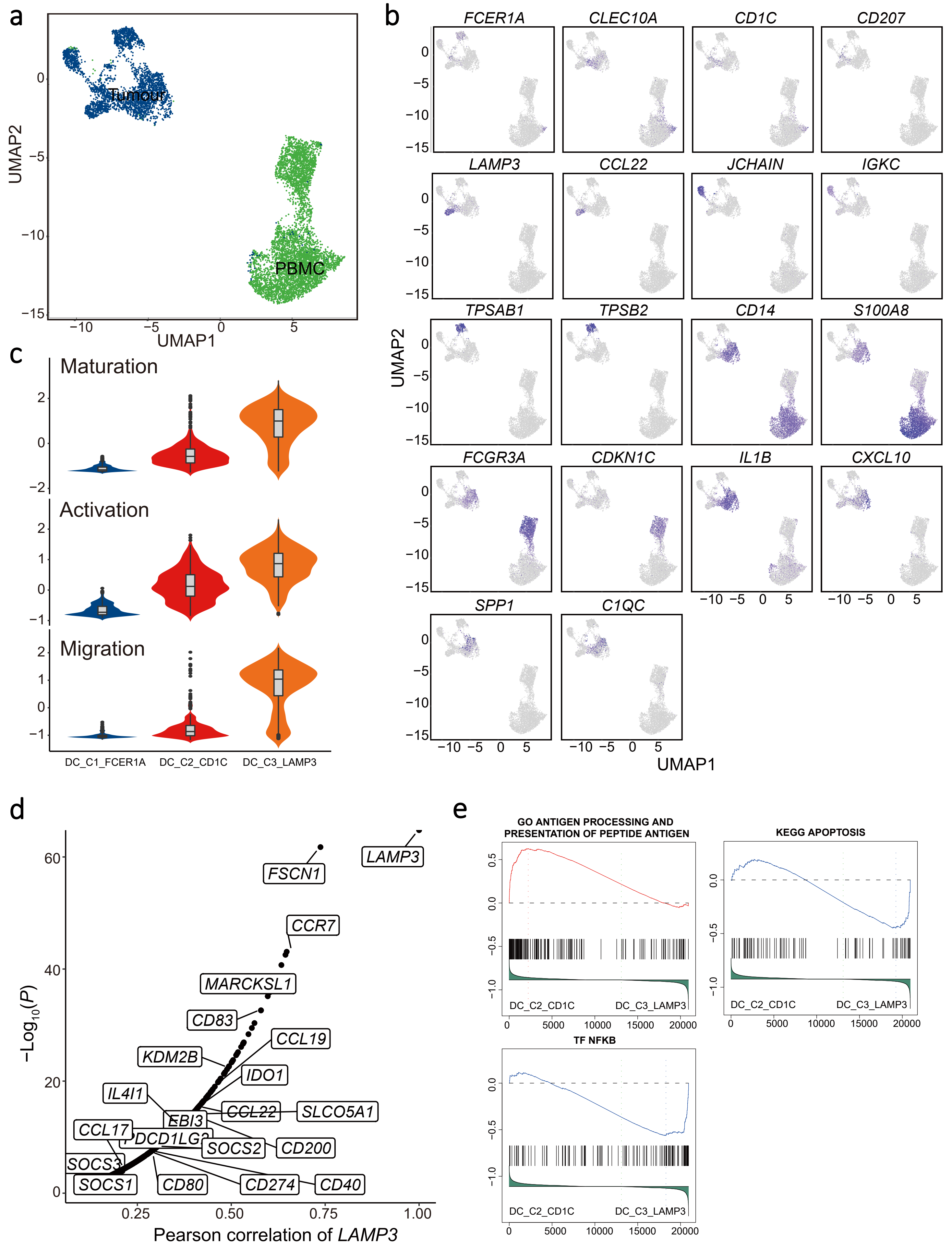

Supplementary Fig. 8 Additional characteristics of myeloid cells.

a UMAP plot showed 8,893 myeloid cells. Each dot represents a cell, coloured according to their origins.

b UMAP plots showed the normalized expression of the marker genes of different myeloid cells. Each dot represents a single cell and the depth of colour from grey to blue represents the expression level from low to high.

c Violin plot showed the maturation, activation and migration scores of each dendritic cell cluster. Box plots inside the violins indicated the quartiles of corresponding score levels (n = 1,134). Endpoints depict minimum and maximum values; centre lines denote median values; whiskers denote 1.5 × the interquartile range; black dots denote each cell. Cell clusters and the signature scores are indicated at the x- and y-axis, respectively.

d Scatter plot showed the genes with significant correlation with *LAMP3* in DC_C3_LAMP3. Selected signature genes are indicated in black boxes. The Pearson correlation coefficients of other genes and *LAMP3* are indicated at the x-axis, and the $-\log_{10}P$ vales are indicated at the y-axis.

e GSEA analyses revealed the pathway activities (on top of each box) between DC_C3_LAMP3 and DC_C2_CD1C.

Supplementary Figure 9

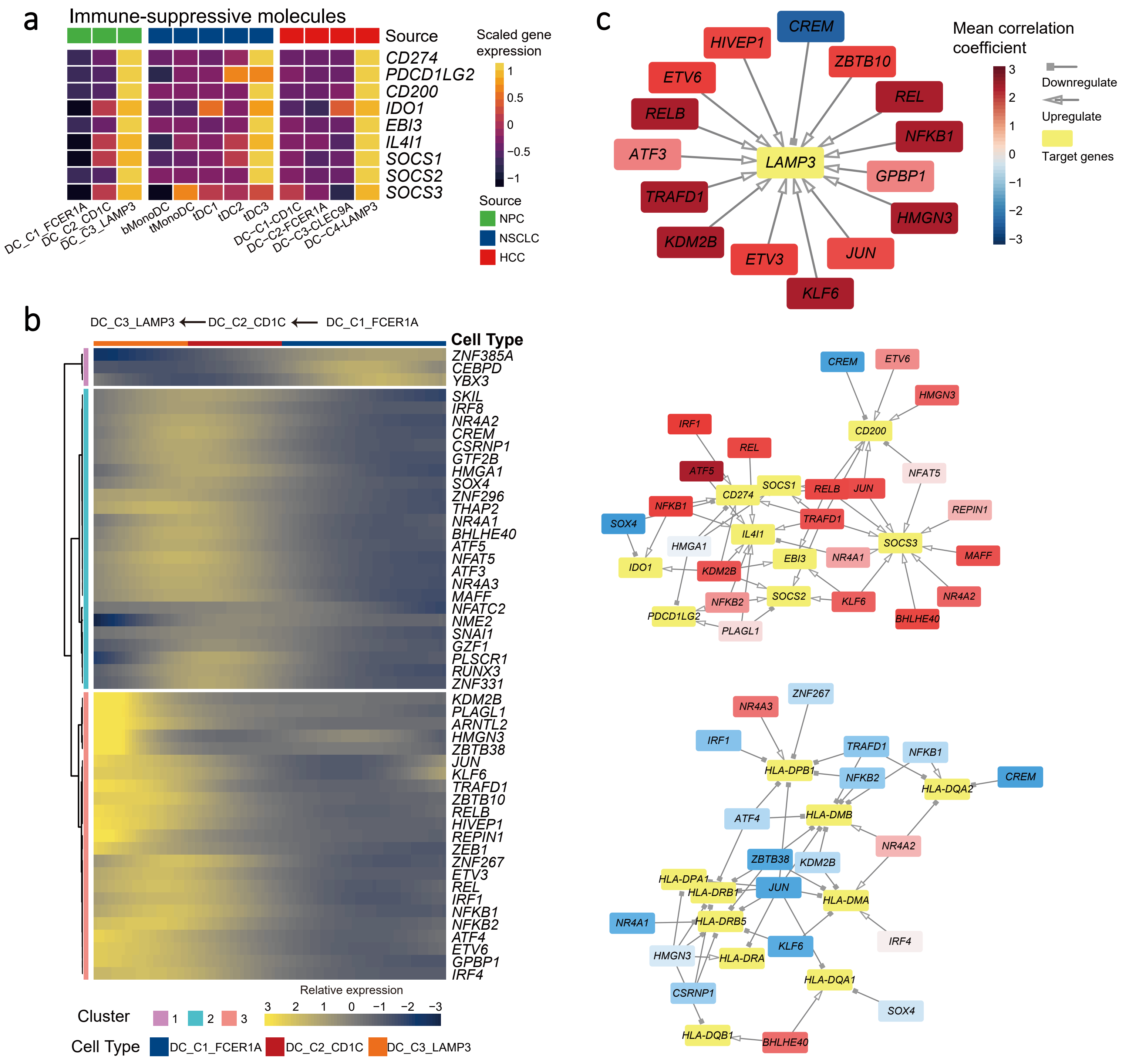

Supplementary Fig. 9 Transcription factor and regulatory network of dendritic cells.

a Heatmap showed the normalized mean expression of cluster markers and immune- suppressive genes (rows) in dendritic cell clusters (columns) derived from multiple cancers. Cancer types were indicated as source on top with different green, blue, and red for NPC, NSCLC (non-small-cell lung cancer), and HCC (hepatocellular carcinoma), respectively. Filled colours from dark blue to yellow represent scaled expression levels from low to high.

b Branched heatmap showed selected transcription factors (rows) of three dendritic cell clusters (columns). Each column line represents a cell, with cell type of origin indicated as blue, red, and orange colours on top for the three clusters. Filled colours from blue to yellow represent scaled expression levels from low to high.

c The regulatory network of transcription factors for *LAMP3* (top panel), immune-suppressive molecules (middle panel), and HLA-II (bottom panel) associated genes in DC_C3_LAMP3 cells. Target genes were coloured in yellow, and the other genes were boxed with colour spectrum from blue to red, indicating mean correlation coefficient from low to high. End arrow type represents regulation effect on the target.

Supplementary Figure 10

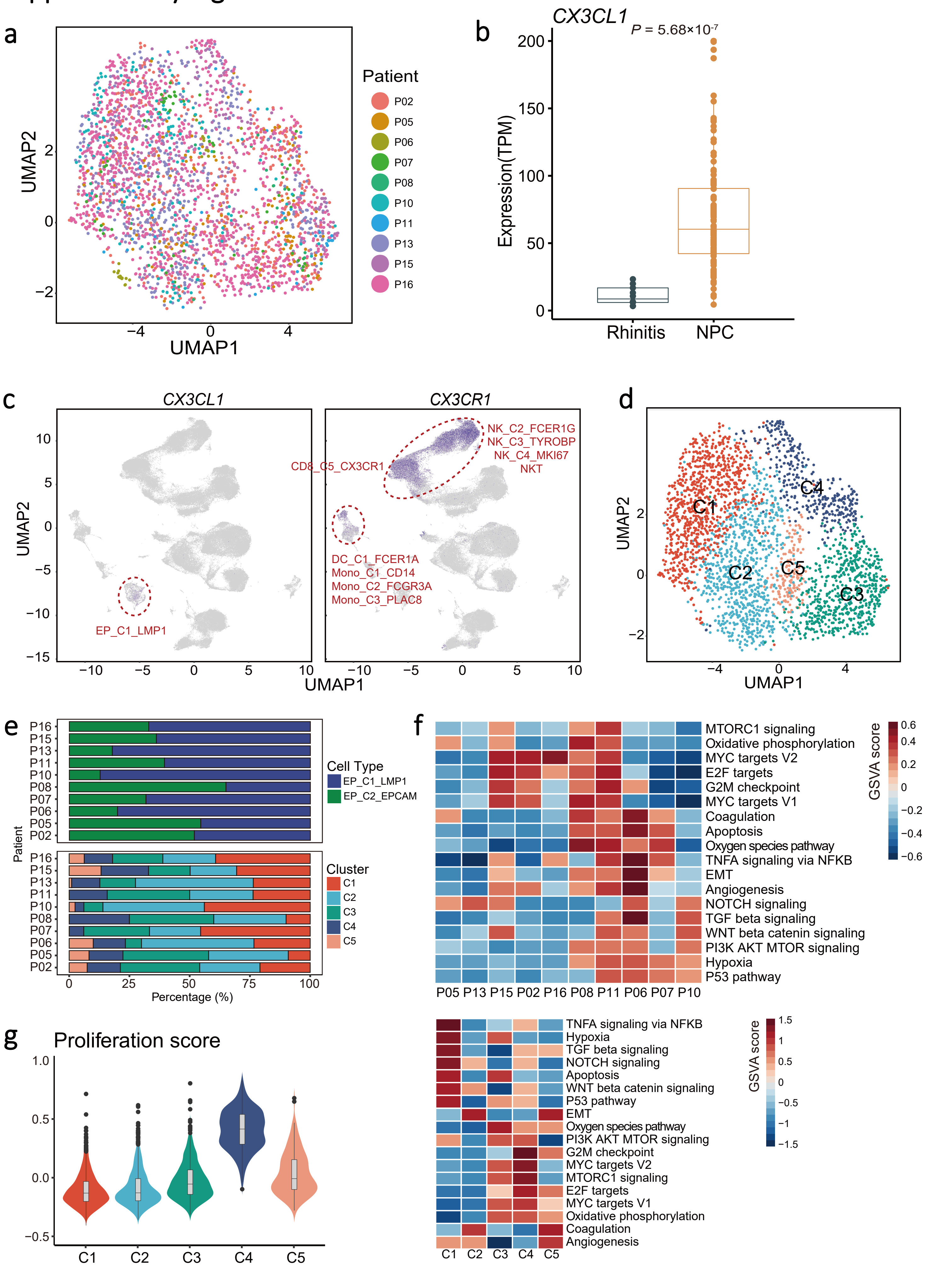

Supplementary Fig. 10 Heterogeneity of malignant cells.

a UMAP plot of all 2,787 malignant NPC cells. Each dot represents a single cell coloured according to the patient of origin.

b Box plot showed gene expression levels (TPM) of *CX3CL1* NPC and non-cancerous tissues. 113 NPC tumour tissues and 10 rhinitis tissues were included (n = 123). Endpoints depict minimum and maximum values; centre lines denote median values; whiskers denote 1.5 × the interquartile range; coloured dots denote each patient. *P* value was calculated using two-sided Wilcoxon test.

c UMAP plots showed the normalized expression of *CX3CL1* (left panel) and *CX3CR1* (right panel) of all 176,447 cells. Each dot represents a single cell and the depth of colour from grey to blue represents low to high expression.

d UMAP plot of 2,787 malignant cells grouped into five cell subtypes. Each dot represents a cell, coloured according to cell subtypes from C1-C5.

e Bar plots showed the variable proportion of malignant cells of different cell clusters. Cell clusters were determined based on EBV^+ and EBV^- (top panel) as well as a further clustering analysis in NPC (bottom panel). Each bar represents a patient with the identifier shown on the left, and cell clusters are shown with different colours.

f Heatmap showed GSVA scores of the gene signatures relevant to carcinogenesis in malignant cells from each patient (top panel) and cluster (bottom panel) as indicated at the bottom. Each row shows a specific pathway. The colour depth from blue to red indicates the GSVA score from low to high.

g Violin plot showed the proliferation scores of each malignant cell cluster in **d.** The scores were calculated by AddModule-Socre function in Seurat (n = 2,787). Box plots inside the violins indicated the quartiles of corresponding score levels. Endpoints depict minimum and maximum values; centre lines denote median values; whiskers denote 1.5 × the interquartile range; black dots denote each cell. Cell clusters and the proliferation score are indicated at the x- and y-axis, respectively.

Supplementary Figure 11

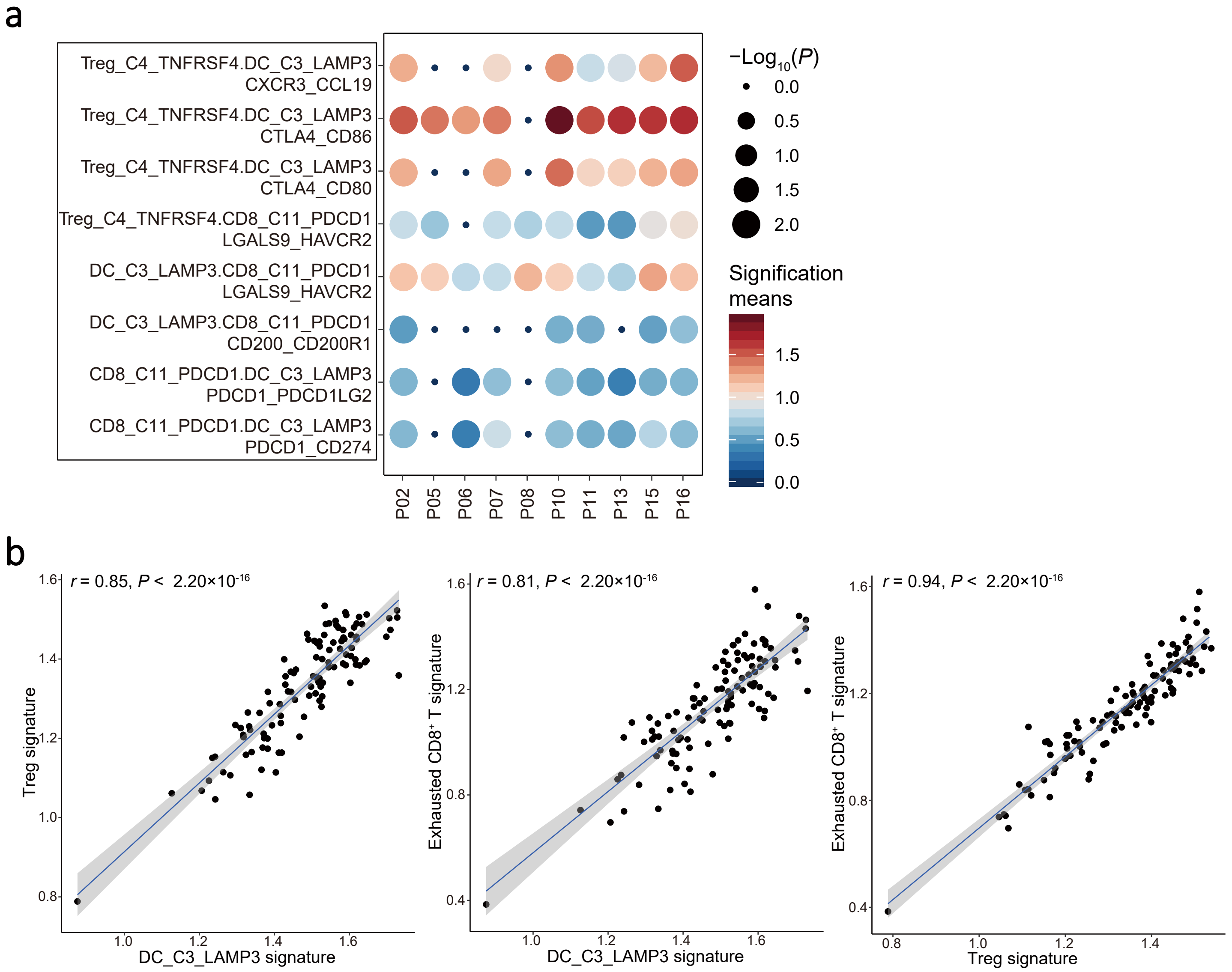

Supplementary Fig. 11 Intercellular communication networks in NPC.

a Dot plots showed selected ligand-receptor interactions (rows) between Treg cells, DC_C3_LAMP3 cells and exhausted CD8^{+} T cells (CD8_C11_PDCD1) for each patient (columns). The ligand-receptor interactions and cell-cell interactions are indicated at columns and rows, respectively. The means of the average expression levels of two interacting molecules are indicated by colour heatmap (right panel), with blue to red representing low to high expression. The $\log_{10}$(*P* values) were indicated by circle size in one-sided permutation test.

b Scatter plots showed the pair-wise correlation of gene signatures among DC_C3_LAMP3 cells, Treg cells, and exhausted CD8^{+} T cells (CD8_C11_PDCD1). 113 NPC samples were included, shown as dots. The *r* values represented Pearson's correlation. The *P* values were calculated by two-sided Pearson correlation analysis and adjusted for multiple comparisons.

Supplementary Figure 12

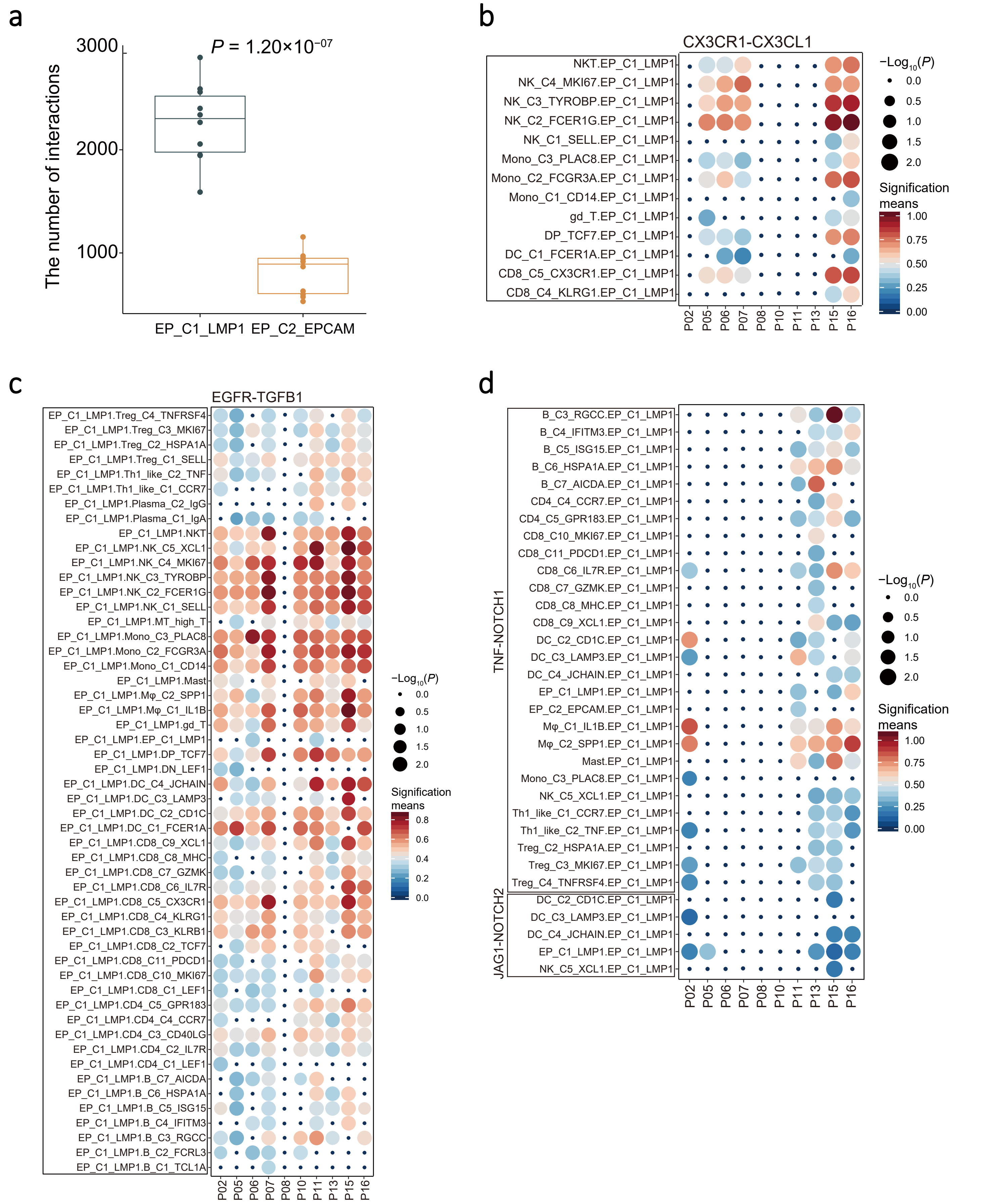

Supplementary Fig. 12 Inter-patient heterogeneity of the interactions in our NPC cohort.

a Box plots showed the number of interactions between malignant cells with other cells in NPC. Cell clusters of EBV^+ (EP_C1_LMP1) and EBV^- (EP_C2_EPCAM) are indicated at the x-axis, and the number of interactions is indicated at the y-axis. Endpoints depict minimum and maximum values; centre lines denote median values; whiskers denote 1.5 × the interquartile range; coloured dots denote each patient. Comparison was made using two-sided paired Student's t-test.

b-d Dot plots showed ligand-receptor interactions of CX3CL1-CX3CR1 (**b**), EGFR-TGFB1 (**c**), and TNF-NOTCH1 and JAG1-NOTCH2 (**d**), and between EBV^+ malignant cells (EP_C1_LMP1) and immune cells (rows) in each patient (columns). The ligand-receptor interactions and cell-cell interactions are indicated at columns and rows, respectively. The means of the average expression levels of two interacting molecules are indicated by colour heatmap (right panel), with blue to red representing low to high expression. The $\log_{10}$(P values) were indicated by circle size in one-sided permutation test. Sample ID is indicated at the bottom of each plot.

Supplementary Figure 13

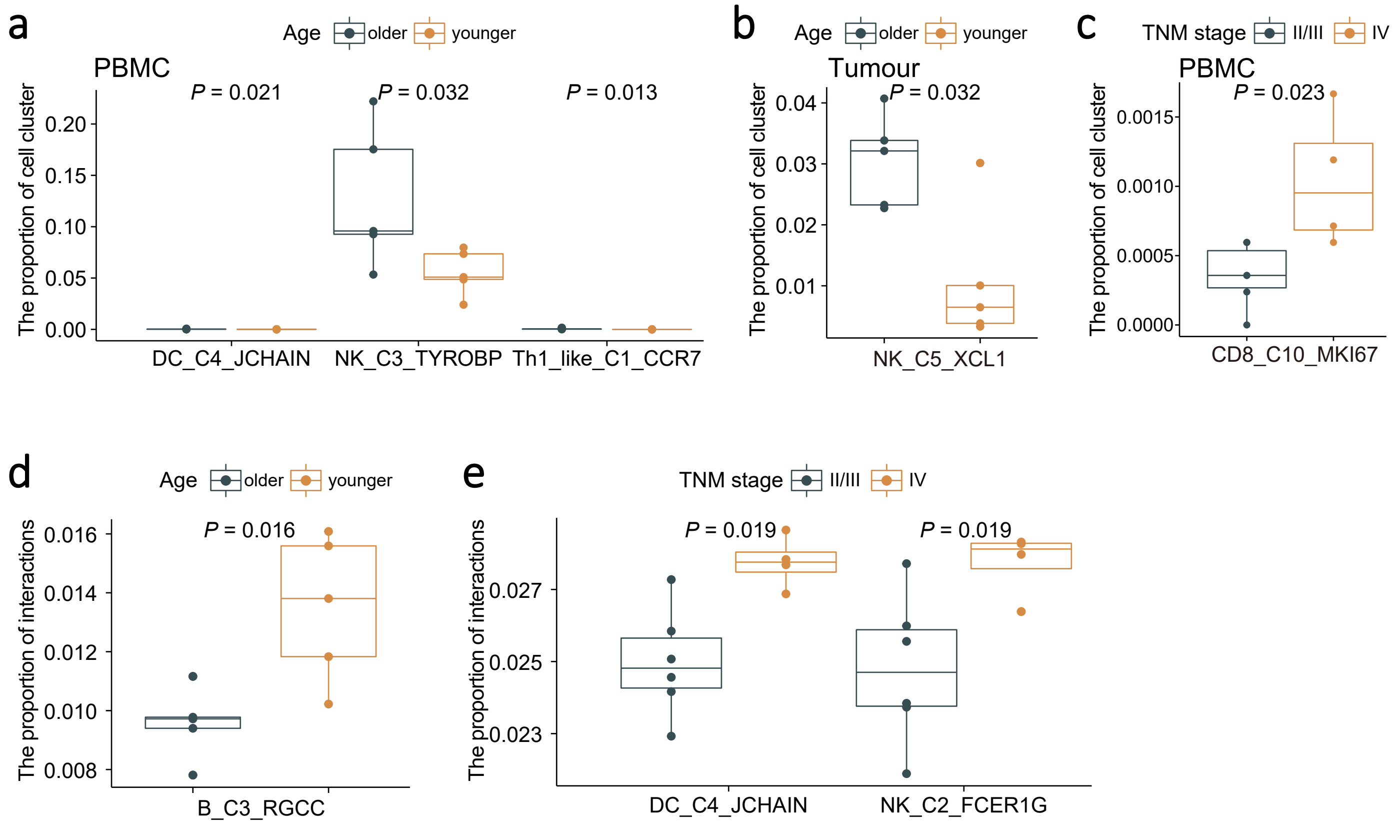

Supplementary Fig. 13 The association of cell composition and cell-cell interaction with patients' characteristics.

a, b Box plots showed the proportions of DC_C4_JCHAIN, NK_C3_TYROBP, and TH1_like_C1_CCR7 in peripheral blood (**a**) and NK_C5_XCL1 in tumour (**b**) from patients with NPC of two age groups (n = 10). Endpoints depict minimum and maximum values; centre lines denote median values; whiskers denote 1.5 × the interquartile range; coloured dots denote each patient.

c Box plots showed the proportions of CD8_C10_MKI67 in peripheral blood from patients with NPC of two TNM staging groups (n = 10). Endpoints depict minimum and maximum values; centre lines denote median values; whiskers denote 1.5 × the interquartile range; coloured dots denote each patient.

d, e Box plots showed the proportion of the cell-cell interactions in B_C3_RGCC, DC_C4_JCHAIN or NK_C2_FCER1G cells among all interactions of each patient with NPC grouped (n = 10) according to their age (**d**) or TNM stage (**e**). Endpoints depict minimum and maximum values; centre lines denote median values; whiskers denote 1.5 × the interquartile range; coloured dots denote each patient.

All above comparisons between groups were made using two-sided Wilcoxon test.

Supplementary Table 1. Clinical characteristics of 10 NPC patients in this study.

Num	SampleID	Pathological diagnosis*	EBERS hybridization	TNM stage*	Metastasis	Collection date	Last follow-up date	Follow-up month	Prognosis of status#
1	P02	Undifferentiated non-keratinized carcinoma	+	T3N2M0, III	Cervical lymph node	2018-6	2019-7	13	0
2	P05	Undifferentiated non-keratinized carcinoma	+	T4N3M1, IVb	Cervical lymph node, shoulder	2018-6	2020-3	21	1
3	P06	Undifferentiated non-keratinized carcinoma	+	T4N3M0, IVa	Cervical lymph node	2018-6	2020-3	21	1
4	P07	Undifferentiated non-keratinized carcinoma	+	T3N2M0, III	Cervical lymph node	2018-7	2020-3	20	1
5	P08	Undifferentiated non-keratinized carcinoma	+	T4N3M0, IVa	Cervical lymph node	2018-7	2020-5	22	1
6	P10	Undifferentiated non-keratinized carcinoma	+	T3N1M0, III	Cervical lymph node	2018-8	2020-3	19	1
7	P11	Undifferentiated non-keratinized carcinoma	+	T3N2M0, III	Cervical lymph node	2018-8	2020-3	19	1
8	P13	Differentiated non-keratinized carcinoma	+	T2N2M0, III	Cervical lymph node	2018-9	Lost to follow-up	NA	NA
9	P15	Undifferentiated non-keratinized carcinoma	+	T2N1M0, II	Cervical lymph node	2018-9	Lost to follow-up	NA	NA
10	P16	Undifferentiated non-keratinized carcinoma	+	T3N3M1, IVb	Cervical lymph node, parotid lymph node	2018-9	2019-1	4	0

*:Pathological diagnosis and TNM stage of NPC were determined according to the 8th edition of the International Union against Cancer (UICC) and American Joint Committee on Cancer (AJCC) staging system.

#:0 stands for deceased, and 1 stands for alive.

Supplementary Table 2. Basical information of single cell RNA sequencing.

Sample	Number of Reads	Saturation
P02_PBMC	400,902,315	85.80%
P02_Tumor	447,735,971	90.60%
P05_PBMC	430,114,076	90.80%
P05_Tumor	443,295,146	88.20%
P06_PBMC	401,681,714	91.80%
P06_Tumor	407,516,376	89.80%
P07_PBMC	409,005,308	91.40%
P07_Tumor	393,318,732	75.90%
P08_PBMC	388,513,428	94.50%
P08_Tumor	437,556,411	89.20%
P10_PBMC	381,407,476	92.90%
P10_Tumor	402,371,890	93.50%
P11_PBMC	411,929,339	92.20%
P11_Tumor	412,740,490	83.50%
P13_PBMC	440,208,784	90.50%
P13_Tumor	387,537,226	92.20%
P15_PBMC	438,406,967	90.70%
P15_Tumor	395,564,848	92.90%
P16_PBMC	408,342,331	92.90%
P16_Tumor	407,237,789	87.10%

Supplementary Table 3. Information of doublet removal.

Sample	Doublet	Single
P02_PBMC	595	11,306
P02_Tumour	604	11,466
P05_PBMC	534	10,153
P05_Tumour	434	8,240
P06_PBMC	453	8,614
P06_Tumour	545	10,348
P07_PBMC	531	10,083
P07_Tumour	590	11,219
P08_PBMC	525	9,980
P08_Tumour	580	11,022
P10_PBMC	506	9,617
P10_Tumour	467	8,869
P11_PBMC	528	10,026
P11_Tumour	333	6,335
P13_PBMC	516	9,804
P13_Tumour	370	7,032
P15_PBMC	451	8,562
P15_Tumour	438	8,319
P16_PBMC	470	8,928
P16_Tumour	437	8,298

Supplementary Table 4. CDR3 shared information of T cells in NPC.

CDR3 a chain	VDJ database	CDR3 b chain	VDJ database	Cell type	Paient	Number
CAAGGGFKTIF	No	CASSPDGDEQFF	No	CD4_C3_CD40LG,CD8_C7_GZMK, CD8_C9_XCL1	P07, P11	10
CAAKEYGNKLVF	No	CAISFDGSGNTIYF	No	CD8_C7_GZMK,CD8_C9_XCL1, CD8_C5_CX3CR1, CD8_C8_MHC	P11, P15	5
CAASTGNNDMRF	No	CASSRQWGNTGELFF	No	CD4_C5_GPR183	P06, P13	2
CAENDDYKLSF	No	CASSQGTTGEAFF	No	CD4_C2_IL7R,CD4_C5_GPR183	P07, P15	5
CAMRERTAGGTSYGKLTF	No	CATSDPGQGAGETQYF	No	CD8_C9_XCL1,CD8_C11_PDCD1, CD8_C7_GZMK,CD8_C8_MHC, CD8_C10_MKI67	P11, P15	21
CAMSPMDTGRRALTF	No	CASSGERDTDTQYF	No	Th1_like_C2_TNF,Treg_C4_TNFRSF4	P06, P08	2
CARWMDSSYKLIF	No	CASSPGGTGANVLTF	No	CD4_C5_GPR183,CD4_C2_IL7R	P02, P10	2
CATDGNTDKLIF	No	CASSFSGANVLTF	EBNA4 (EBV)	CD4_C3_CD40LG,Th1_like_C1_CCR7	P05, P16	2
CATVEAAGNKLTF	No	CASSLSGYGYTF	No	Th1_like_C1_CCR7,Th1_like_C2_TNF	P08, P11	2
CAVISRGTGFQKLVF	No	CASSQGGYEQYF	No	CD8_C11_PDCD1,CD8_C10_MKI67, CD8_C8_MHC, CD8_C9_XCL1, CD8_C4_KLRG1,CD8_C5_CX3CR1	P06, P10	11
CAVKANQAGTALIF	No	CASSLGGTQYF	No	CD8_C11_PDCD1	P05, P06	2
CAVRGTGTASKLTF	ELAGIGILTV (MLANA)	CSVRQGGNEQFF	No	CD8_C7_GZMK,CD8_C4_KLRG1, CD8_C5_CX3CR1	P08, P11	13
CAVRGTGTASKLTF	ELAGIGILTV (MLANA)	CSVRAGGNEQFF	No	CD8_C7_GZMK,CD8_C4_KLRG1, CD8_C5_CX3CR1	P08, P11	35
CAVTNDYKLSF	No	CASSYSPGRDEQFF	No	CD4_C5_GPR183,CD8_C5_CX3CR1, CD8_C7_GZMK	P11, P15	11

Supplementary Table 5. Genes used as markers of different cell features and the references that justified their use.

Features	Markers
Tissue resident T cell	CD69, CD103[1], BHLHE40[2],
Naïve B cell	IGHM[3], IGHD[4]
Dendritic cell maturation	LAMP3, MARCKSL1, IDO1[5], UBD[6]
Dendritic cell activation	CD80, CD83, CD40[7]
Dendritic cell migration	CCR7, FSCN1, SLCO5A1[8]
Dendritic cell immune-supressive related genes	CD274, PDCD1LG2, CD200[9], EBI3, IDO1, IL4I1[10],SOCS1, SOCS2[11], SOCS3

Reference:

1. Liu Y, Ma C, Zhang N. Tissue-Specific Control of Tissue-Resident Memory T Cells. *Crit Rev Immunol* **38**, 79-103 (2018).
2. Li C*, et al.* The Transcription Factor Bhlhe40 Programs Mitochondrial Regulation of Resident CD8(+) T Cell Fitness and Functionality. *Immunity* **51**, 491-507 e497 (2019).
3. Kong F*, et al.* Abnormal phenotypic features of IgM+B cell subsets in patients with chronic hepatitis C virus infection. *Exp Ther Med* **14**, 1846-1852 (2017).
4. Nechvatalova J, Bartol SJW, Chovancova Z, Boon L, Vlkova M, van Zelm MC. Absence of Surface IgD Does Not Impair Naive B Cell Homeostasis or Memory B Cell Formation in IGHD Haploinsufficient Humans. *J Immunol* **201**, 1928-1935 (2018).
5. Harden JL, Egilmez NK. Indoleamine 2,3-dioxygenase and dendritic cell tolerogenicity. *Immunol Invest* **41**, 738-764 (2012).
6. Hole CR*, et al.* Induction of memory-like dendritic cell responses in vivo. *Nat Commun* **10**, 2955 (2019).
7. Mihret A, Mamo G, Tafesse M, Hailu A, Parida S. Dendritic Cells Activate and Mature after Infection with Mycobacterium tuberculosis. *BMC Res Notes* **4**, 247 (2011).
8. Miller JC*, et al.* Deciphering the transcriptional network of the dendritic cell lineage. *Nat Immunol* **13**, 888-899 (2012).
9. Rygiel TP*, et al.* CD200-CD200R signaling suppresses anti-tumor responses independently of CD200 expression on the tumor. *Oncogene* **31**, 2979-2988 (2012).
10. Maier B*, et al.* A conserved dendritic-cell regulatory program limits antitumour immunity. *Nature* **580**, 257-262 (2020).
11. Nirschl CJ*, et al.* IFNgamma-Dependent Tissue-Immune Homeostasis Is Co-opted in the Tumor Microenvironment. *Cell* **170**, 127-141 e115 (2017).
