# Supplementary material for: Tumour heterogeneity and intercellular networks of nasopharyngeal carcinoma at single cell resolution
Source: Nat Commun. 2021 Feb 2;12:741. doi: 10.1038/s41467-021-21043-4 (PMC7854640; doi:10.1038/s41467-021-21043-4)
Supplement: Supplementary file 3 — Description of Additional Supplementary Files [file 41467_2021_21043_MOESM3_ESM.pdf]

Description of Additional Supplementary Files

File Name: Supplementary Data 1
Description: Cell number and UMI information of each cluster and patient.

File Name: Supplementary Data 2
Description: Differentially expressed genes in NK, T, B, myeloid, and malignant cells

File Name: Supplementary Data 3
Description: Signature score related genes.

File Name: Supplementary Data 4
Description: TCR information of T cells in NPC.

File Name: Supplementary Data 5
Description: Transcription factors regulatory network in LAMP3^{+} DCs.

File Name: Supplementary Data 6
Description: Significant ligand-receptor interaction pairs between cell types in NPC.

File Name: Supplementary Data 7
Description: Description: Cluster signature genes (Top Rank 200).
