# Supplementary material for: Tumour heterogeneity and intercellular networks of nasopharyngeal carcinoma at single cell resolution
Source: Nat Commun. 2021 Feb 2;12:741. doi: 10.1038/s41467-021-21043-4 (PMC7854640; doi:10.1038/s41467-021-21043-4)
Supplement: Supplementary file 11 — Reporting Summary [file 41467_2021_21043_MOESM11_ESM.pdf]

nature research

Corresponding author(s): Jin-Xin Bei

Last updated by author(s): Nov 27, 2020

Reporting Summary

Nature Research wishes to improve the reproducibility of the work that we publish. This form provides structure for consistency and transparency in reporting. For further information on Nature Research policies, see our Editorial Policies and the Editorial Policy Checklist.

Statistics

For all statistical analyses, confirm that the following items are present in the figure legend, table legend, main text, or Methods section.

n/a	Confirmed	
☐	☒	The exact sample size (*n*) for each experimental group/condition, given as a discrete number and unit of measurement
☐	☒	A statement on whether measurements were taken from distinct samples or whether the same sample was measured repeatedly
☐	☒	The statistical test(s) used AND whether they are one- or two-sided<br>*Only common tests should be described solely by name; describe more complex techniques in the Methods section.*
☐	☒	A description of all covariates tested
☐	☒	A description of any assumptions or corrections, such as tests of normality and adjustment for multiple comparisons
☐	☒	A full description of the statistical parameters including central tendency (e.g. means) or other basic estimates (e.g. regression coefficient) AND variation (e.g. standard deviation) or associated estimates of uncertainty (e.g. confidence intervals)
☐	☒	For null hypothesis testing, the test statistic (e.g. *F*, *t*, *r*) with confidence intervals, effect sizes, degrees of freedom and *P* value noted<br>*Give P values as exact values whenever suitable.*
☒	☐	For Bayesian analysis, information on the choice of priors and Markov chain Monte Carlo settings
☒	☐	For hierarchical and complex designs, identification of the appropriate level for tests and full reporting of outcomes
☐	☒	Estimates of effect sizes (e.g. Cohen's *d*, Pearson's *r*), indicating how they were calculated

Our web collection on statistics for biologists contains articles on many of the points above.

Software and code

Policy information about availability of computer code

Data collection	Illumina HiSeq system to generate sequencing data.
Data analysis	Flow cytometry data was analysed using BD FACS Diva version 8.<br>Transcriptome data was analysed using bcl2fastq (https://support.illumina.com/sequencing/sequencing_software/bcl2fastq-conversion-software/downloads.html; version 2.19.0.316), CellRanger (https://support.10xgenomics.com/single-cell-gene-expression/software/pipelines/latest/installation; version 3.0.1), Seurat (https://satijalab.org/Seurat;version 2.3.4), DoubletFinder (https://github.com/chris-mcginnis-ucsf/DoubletFinder; 2.0.1), Monocle (http://cole-trapnell-lab.github.io/monocle-release/docs; version 2.8), STARTRAC (https://github.com/Japrin/STARTRAC; version 0.1), GSEA (https://www.gsea-msigdb.org/gsea/downloads.jsp; version 3.0), GSVA (https://github.com/rcastelo/GSVA; version 1.30.0), Metascape (https://metascape.org/gp/index.html), ARACNe-AP (https://github.com/califano-lab/ARACNe-AP), inferCNV (https://github.com/broadinstitute/infercnv; version 1.2.1), CellPhoneDB (https://github.com/Teichlab/cellphonedb; version 2.06), Bowtie2 (https://github.com/BenLangmead/bowtie2; version 2.3.4.2), and HISAT2 (https://github.com/DaehwanKimLab/hisat2; version 2.1.0).In-house scripts are available at https://github.com/bei-lab/scRNA-of-NPC.

For manuscripts utilizing custom algorithms or software that are central to the research but not yet described in published literature, software must be made available to editors and reviewers. We strongly encourage code deposition in a community repository (e.g. GitHub). See the Nature Research guidelines for submitting code & software for further information.

Data

Policy information about availability of data

All manuscripts must include a data availability statement. This statement should provide the following information, where applicable:

- Accession codes, unique identifiers, or web links for publicly available datasets
- A list of figures that have associated raw data
- A description of any restrictions on data availability

The raw sequence data reported in this study have been deposited in the Genome Sequence Archive (Genomics, Proteomics & Bioinformatics 2017) in National Genomics Data Center (Nucleic Acids Res 2020), Beijing Institute of Genomics (China National Center for Bioinformation), Chinese Academy of Sciences, under accession number HRA000159 (accessible at http://bigd.big.ac.cn/gsa-human) and GEO dataset under the accession number GSE162025. The key data in this study has also been deposited in the Research Data Deposit (RDDB2020000980; http://www.researchdata.org.cn/). Other datasets used can be downloaded from NCBI GEO under the accession numbers GSE132465, GSE146771, GSE99254, GSE140228, GSE127465, GSE102349, and GSE121600, Genome Sequence Achieve under the accession numbers CRA001160, and the URL https://gbiomed.kuleuven.be/scRNAseq-NSCLC. The remaining data are available within the Article, Supplementary Information or available from the authors upon request.

Field-specific reporting

Please select the one below that is the best fit for your research. If you are not sure, read the appropriate sections before making your selection.

[x] Life sciences [ ] Behavioural & social sciences [ ] Ecological, evolutionary & environmental sciences

For a reference copy of the document with all sections, see nature.com/documents/nr-reporting-summary-flat.pdf

Life sciences study design

All studies must disclose on these points even when the disclosure is negative.

Sample size	Sample size for 10x Genomics scRNA-seq was determined by the availability of patient samples.<br>No statistical tests were performed for sample size calculation but it was sufficient for this proof-of-concept study. The exact number of samples used per figure is informed in each figure. In the whole manuscript, they are: 10 endoscopic biopsy tissues of nasopharyngeal carcinoma (fresh)
Data exclusions	All criteria for data exclusion were pre-established. We removed doublets in each sample by R package "DoubletFinder", with an expected doublet rate of 0.05 and default parameters used otherwise. Next, any cells were removed for which had either less than 101 UMIs, or expression of less than 501 genes, or over 15% UMIs linked to mitochondrial genes.
Replication	Multiplex IF and IHC staining assays were confirmed in at least three biological replicates.
Randomization	The patients with nasopharyngeal carcinoma were recruited randomly in this study.
Blinding	Investigators were blinded to patient identity. Tumour biopsy was collected and sent for downstream procedures with coded Sample_ID.

Reporting for specific materials, systems and methods

We require information from authors about some types of materials, experimental systems and methods used in many studies. Here, indicate whether each material, system or method listed is relevant to your study. If you are not sure if a list item applies to your research, read the appropriate section before selecting a response.

Materials & experimental systems

n/a	Involved in the study	
[ ]	[x]	Antibodies
[x]	[ ]	Eukaryotic cell lines
[x]	[ ]	Palaeontology and archaeology
[x]	[ ]	Animals and other organisms
[ ]	[x]	Human research participants
[x]	[ ]	Clinical data
[x]	[ ]	Dual use research of concern

Methods

n/a	Involved in the study	
[x]	[ ]	ChIP-seq
[ ]	[x]	Flow cytometry
[x]	[ ]	MRI-based neuroimaging

Antibodies

Antibodies used	All antibodies were commercially purchased and included: anti-EPCAM (rabbit; Abcam; Cat. no. ab71916), anti-LMP1 (mouse; Abcam; Cat. no. ab78113), anti-CD3 (rabbit; Abcam; Cat. no. ab135372), anti-CD4 (rabbit; Abcam; Cat. no. ab133616), anti-CD8A (mouse; CST; Cat. no. CST70306), anti-FOXP3 (mouse; Abcam; Cat. no. ab22510), anti-CD80 (mouse; R&D Systems; Cat. no. MAB140), anti-

PD1 (mouse; CST; Cat. no. CST43248), anti-PD-L1 (rabbit; CST; Cat. no. CST13684), anti-CTLA4 (rabbit; Abcam; Cat. no. ab237712), anti-Rabbit IgG (Goat; Servicebio; Cat. No. GB21303), and anti-Mouse IgG (Goat; Servicebio; Cat. No. GB21301).

Validation

All the antibodies used in this study were commercial antibodies, with validation procedures described on the following sites of the manufacturers:
anti-EPCAM (rabbit; Abcam; Cat. no. ab71916)
https://www.abcam.com/epcam-antibody-ab71916.html
anti-LMP1 (mouse; Abcam; Cat. no. ab78113)
https://www.abcam.com/ebv-latent-membrane-protein-1-antibody-cs-1-4-ab78113.html
anti-CD3 (rabbit; Abcam; Cat. no. ab135372)
https://www.abcam.com/cd3-antibody-sp162-ab135372.html
anti-CD4 (rabbit; Abcam; Cat. no. ab133616)
https://www.abcam.com/cd4-antibody-epr6855-ab133616.html
anti-CD8A (mouse; CST; Cat. no. CST70306)
https://www.cellsignal.com/products/primary-antibodies/cd8a-c8-144b-mouse-mab-ihc-specific/70306
anti-FOXP3 (mouse; Abcam; Cat. no. ab22510)
https://www.abcam.com/foxp3-antibody-mabcam-22510-ab22510.html
anti-CD80 (mouse; R&D Systems; Cat. no. MAB140)
https://www.rndsystems.com/products/human-b7-1-cd80-antibody-37711_mab140
anti-PD1 (mouse; CST; Cat. no. CST43248)
https://www.cellsignal.com/products/primary-antibodies/pd-1-eh33-mouse-mab-ihc-specific/43248
anti-PD-L1 (rabbit; CST; Cat. no. CST13684)
https://www.cellsignal.com/products/primary-antibodies/pd-l1-e1l3n-xp-rabbit-mab/13684
anti-CTLA4 (rabbit; Abcam; Cat. no. ab237712)
https://www.abcam.com/ctla4-antibody-cal49-ab237712.html
anti-Rabbit IgG (Goat; Servicebio; Cat. No. GB21303)
https://www.servicebio.cn/goodsdetail?id=253
anti-Mouse IgG (Goat; Servicebio; Cat. No. GB21301)
https://www.servicebio.cn/goodsdetail?id=251

Human research participants

Policy information about studies involving human research participants

Population characteristics

10 patients with pathological NPC diagnosis were enrolled in this study. None of the patients had received prior treatment. Detailed information can be found in the Patient recruitment and sample collection section of Methods and Supplementary Table 1.

Recruitment

All donors are recruited and managed by Sun Yat-sen University Cancer Centre, avoiding the selection of poorly clinically characterized volunteers.

Ethics oversight

The medical ethics committee of the Institutional Review Board of Sun Yat-sen University Cancer Center.

Note that full information on the approval of the study protocol must also be provided in the manuscript.

Flow Cytometry

Plots

Confirm that:

- [x] The axis labels state the marker and fluorochrome used (e.g. CD4-FITC).
- [x] The axis scales are clearly visible. Include numbers along axes only for bottom left plot of group (a 'group' is an analysis of identical markers).
- [x] All plots are contour plots with outliers or pseudocolor plots.
- [x] A numerical value for number of cells or percentage (with statistics) is provided.

Methodology

Sample preparation

Tumour tissues were cut into small pieces, followed by collagenase digestion and filter by 40-µm cell-strainer. PBMCs were isolated using leukocyte separation solution. After lysing red blood cells and washing twice with DPBS, the dissociated cells from tumour and blood peripheral were resuspended in 0.04% BSA.

Instrument

BD FACSAria III, BD Biosciences, USA

Software

BD FACS Diva version 8

Cell population abundance

Up to 10^6 single viable cells were sorted from each PBMC and tumour sample. Purity was typically > 98%.

Gating strategy

All samples were gated on singlets and PI (indicating apoptotic cells or fractions). The gating strategy for phenotyping is

shown in Supplementary Figure 1b.

- [x] Tick this box to confirm that a figure exemplifying the gating strategy is provided in the Supplementary Information.
